# Microbial Primer: Bayesian learning of traits from microbial time series data

**DOI:** 10.1099/mic.0.001739

**Published:** 2026-08-10

**Authors:** Raunak Dey, Robert Beach, Kennedi M. Hambrick, Ioannis Sgouralis, Paul Frémont, David Demory, Eric Carr, Stephen J. Beckett, Joshua S. Weitz, David Talmy

**Affiliations:** 1 Department of Physics, University of Maryland, College Park, MD, USA; 2 Department of Microbiology, University of Tennessee Knoxville, Knoxville, TN, USA; 3 Department of Mathematics, University of Tennessee Knoxville, Knoxville, TN, USA; 4 Department of Biology, University of Maryland, College Park, MD, USA; 5 Laboratoire de Biodiversité et Biotechnologies Microbiennes (LBBM), Observatoire Océanologique, Sorbonne Université, CNRS, UMR8176, F-66650 Banyuls-sur-Mer, France; 6 Institute for Health Computing, University of Maryland, North Bethesda, MD, USA

**Keywords:** ecosystem modeling, Bayesian inference, population dynamics, microbial growth kinetics, Monte Carlo Markov Chain, uncertainty quantification

## Abstract

Mathematical models are increasingly used to infer traits, interactions and functional dynamics of microbial systems. One common example is a rate-based ordinary differential equation model parameterized with microbial traits. However, fitting such models with associated parameters to data requires a principled approach to extract information from time series while accounting for prior knowledge and measurement noise. These principles often remain implicit and not necessarily well defined. Here, we make the implicit, explicit: introducing Bayesian inference of ecological models for microbial time series, including three detailed case studies of algal population dynamics that follow a birth-death process. Complementing this primer, we provide an online tutorial on Bayesian inverse modelling with cross-programming language support via Python (PyMC) and Julia (Turing). By connecting theory, code, data and a series of hands-on educational modules, this primer aims to bring the utility of Bayesian learning to the broader microbial ecology research community.

## Code and data availability

All datasets and code are available via GitHub: https://github.com/B2-Bayesian-for-Biology/MCMCwithODEs_primer and Zenodo: https://doi.org/10.5281/zenodo.17792668. The educational resources for this project are available at our webpage:https://b2-bayesian-for-biology.github.io/MCMCwithODEs_primer

## Introduction

Microbial traits shape community structure, ecosystem function and global biogeochemical cycles. Traits include any property of an organism describing its size, shape, composition or function. Knowledge of traits that define population growth and decline allows ecological models to quantitatively recapitulate microbial dynamics both *in vitro* and *in situ*. These traits – such as growth rates, nutrient affinities or mortality parameters – can be directly derived from population time-series data by curve fitting. However, inferred trait values are sensitive to assumptions about the statistical properties of the data, model structures and methods to fit models to data. This primer aims to elucidate Bayesian methods for imputing microbial traits in a focal context: batch culture experiments of algal population dynamics. However, the methodologies and case studies established in this primer are generally applicable to estimating microbial traits from microbial time series across aquatic, terrestrial and host-associated habitats.

Bayesian inference provides a principled approach to link mechanistic models with data and understand ecological dynamics in lab or field settings [1–4]. Bayesian methods combine prior knowledge with observed data to generate probability distributions of trait values, making uncertainty explicit. The Bayesian inference framework has another advantage: it provides a means to integrate information hierarchically, so that information from experiments that isolate components of biological systems may be combined. Here, we focus on identifying key decision points in Bayesian inference as a means to facilitate use by microbiologists rather than enumerating all choices that can be found in other comprehensive reviews [5–8]. Advances in probabilistic programming via software packages, such as PyMC, Stan, Turing and others, make it possible to apply robust sampling techniques for parameter inference, including Markov Chain Monte Carlo (MCMC). These tools have been widely adopted in diverse fields from epidemiology and microscopy to astronomy, underscoring their generality and reliability – and we advocate for more microbiologists to integrate these methods into their basic practice.

This primer introduces Bayesian learning of microbial traits from time-series data via a series of examples that are intended to serve as templates for extension to more complex studies. We outline key decision points in model construction, illustrate applications with case studies and provide starter code across languages (using PyMC in Python and Turing in Julia) via our online resources at https://b2-bayesian-for-biology.github.io/MCMCwithODEs_primer. Additional technical details are provided in the Supplementary Information. Our goal is to help microbiologists move beyond black-box parameter fitting toward transparent, uncertainty-aware inference that strengthens ecological interpretation and predictive modelling.

## What is Bayesian inference?

Bayesian inference balances prior information, such as the knowledge of trait parameters, with new observations, such as recorded population dynamics, to yield an updated view of biologically relevant parameters. The goal of Bayesian inference is to quantify the probability of all possible parameter values given the observed data and the model. Mathematically, this is expressed as a probability distribution, known as the posterior: *P*(θ | D), where θ is the parameter set and D is the observed data. The posterior *P*(θ | D) is the updated probability distribution for a model’s parameters after taking new evidence into account – it is a full uncertainty quantification for trait parameters (e.g. growth rates, nutrient affinities, adsorption, etc.).

Bayesian inference combines three components to infer the posterior. The prior distribution, *P*(*θ*), is a probability distribution that represents our initial belief or knowledge about parameters *θ* before any data has been observed. The prior distribution is usually informed by expert knowledge or independent experimental observation. The likelihood function, *L*(*D | θ*), measures how well a model explains the observed data by calculating the probability of observing those data given different values of the model parameters. Finally, *P*(*D*) is the probability of the particular set of observations. Bayes’ theorem relates these quantities as follows:


$$P(\theta |D)=\frac{L(D|\theta )\;P(\theta )}{P(D)}$$

In principle, any Bayesian analysis should define and quantify the likelihood, *L*(*D | θ*), the priors, *P*(*θ*) and the probability of the data, *P*(*D*). But how are these known?

A key simplification is to recognize that the probability of the data, *P*(*D*), is independent of the parameters. The total posterior probability summed over all parameters *P*(*θ | D*) is one. As a result, *P*(*D*) is not explicitly calculated and rather, acts as a proportionality constant, that yields:


P(θ∣D)∝L(D∣θ)P(θ)

For ordinary differential equation (ODE) models of microbial dynamics, the likelihood quantifies deviations between observed time series and model trajectories. In practice, under the assumption that error scales with the magnitude of measurement, this is usually calculated as a function of the error sum of squared differences between log-transformed data and model predictions.

The practitioner is at liberty to define their own prior probability distribution. In the absence of prior information, a uniform distribution is often assumed; in that case, the results from the Bayesian analysis resemble non-Bayesian (frequentist) interval estimates.

Each of the probability distributions in Equation 2 can in principle be formulated analytically, but this is usually impractical for multivariate ODEs with many parameters. The most practical way to generate representations of these probability distributions is to ‘sample’ many different candidate parameter sets and calculate their associated probabilities. Modern software packages, such as PyMC, Stan, Turing and others do this via MCMC based sampling algorithms.

Samplers work by generating a sequence of parameter values that collectively approximate the posterior distribution starting from an initial parameter set. Samplers use various proposal rules where new parameters are proposed based on the current parameter set. The parameters are accepted or rejected with a probability that depends on how well they explain the data relative to the previous parameter(s). Parameter sets that increase posterior probability are always accepted, whereas parameter sets with lower probability may still be accepted. The goal of these samplers is to exhaustively sample the whole parameter space efficiently, without getting trapped in a single region of high probability. Interested readers can find more information about different samplers from the reading list.

## Why Bayesian?

Bayesian statistics often complement frequentist statistics with informative priors. In Fig. S1, we show that for estimating growth rates, Bayesian inference yields comparable mean results to common frequentist techniques, such as least squares, maximum likelihood estimation and bootstrapping (Methods in Supplementary Information S5.) In simpler cases, it may often be preferable to opt for these simpler approaches. As models become more complicated, Bayesian samplers have advantages that are particularly valuable in the context of ODE systems. In sum, Bayesian samplers provide benefits through:

1. **Integration of prior knowledge**. Integrating informative priors for one parameter can reduce uncertainty for other parameters. We will demonstrate this capability in Section 1 by showing how literature-informed death rate priors lead to revised estimates of *Phaeocystis globosa* growth rates.
2. **Uncertainty quantification**. A related idea is the ability of Bayesian approaches to explicitly quantify uncertainty that arises due to measurement error and natural variation, as well as uncertainty introduced by model structure. The latter issue can be especially important in the context of ODE systems with the potential to yield similar dynamics for different parameter combinations. Parameter uncertainty quantification is central to all three case studies and enabled by our use of a Bayesian framework.
3. **Model flexibility with new data**. In Bayesian inference, one can update the posterior *sequentially* by redefining the likelihood with new data and treating older estimates as priors, an ability we demonstrate in Section 2 by utilizing cellular death data to improve estimates of *Emiliania huxleyi* growth and death rates.
4. **Explicit covariance structure**. Bayesian sampling reveals posterior correlations between parameters, a key indicator of uncertainty associated with model structure and non-identifiability. In Section 3, we demonstrate how Bayesian informed model reformulation enables quantification of *E. huxleyi* nutrient quotas.
5. **Extension across models and data types**. For complex multivariate models, where fitting multiple parameters simultaneously is challenging, Bayesian inference can *hierarchically* integrate parameter estimates from simpler models. The last two case studies demonstrate this ability, moving from a simple logistic model to a logistic model with an explicit death rate and to a Monod growth model.

Importantly, many of these advantages are not unique to Bayesian inference per se, but rather arise from the broader ability to systematically explore parameter spaces, incorporate constraints and quantify uncertainty.

## Case studies

**Fig. 1.**
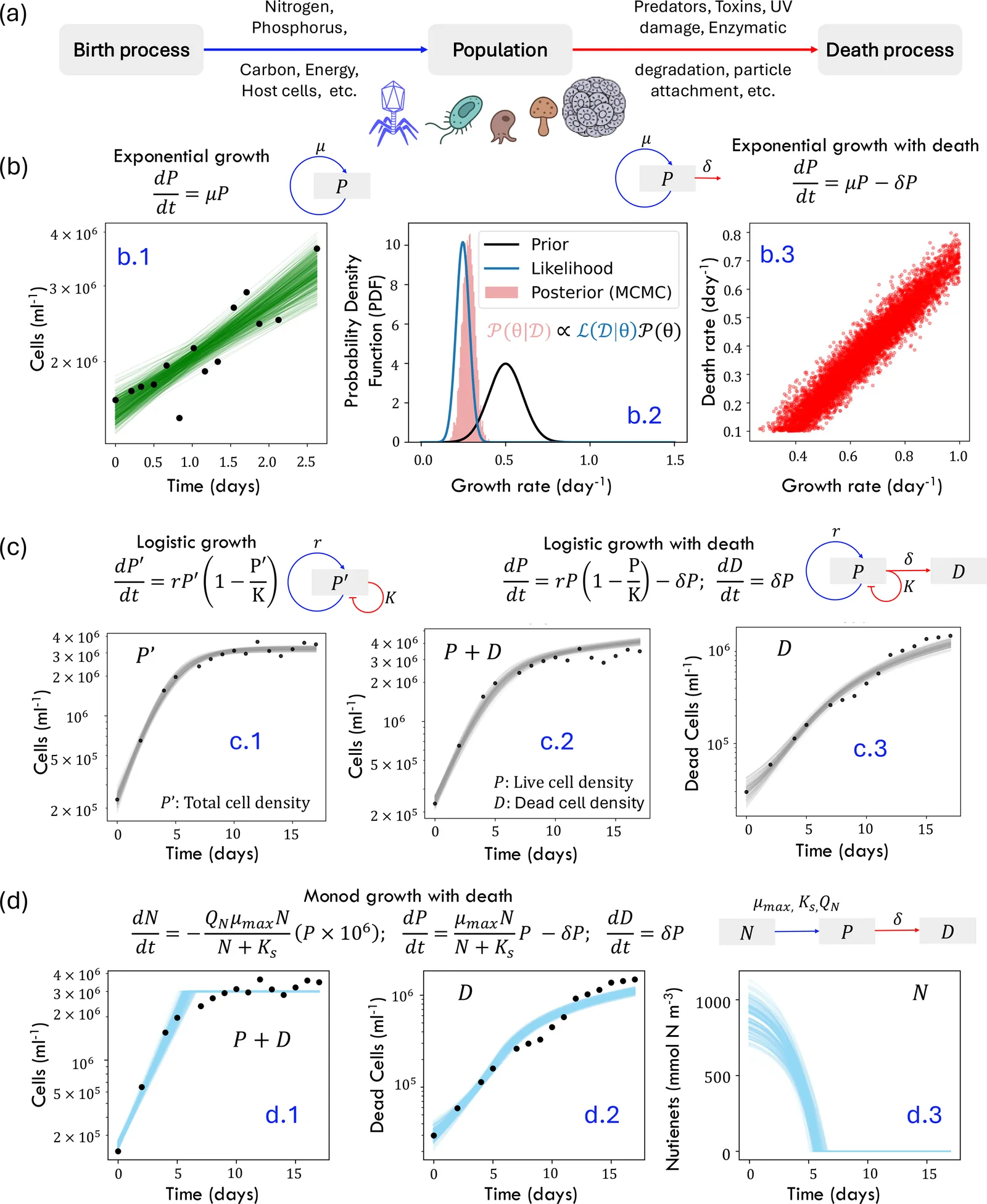
Bayesian fitting for microbial dynamics. (a) Birth-death process: Population dynamics and community structure are a balance of growth and loss processes. They can be modelled by rate-based differential equations, where gain and loss terms due to other agents in the system dictate how the population changes over time. (b) Exponential growth model: (b.1) shows a single run of *P*. globosa population growth over time; data in solid dots and model solutions with parameters randomly drawn from posterior distributions are shown in green. (b.2) Demonstration of Bayes’ rule for estimating the exponential growth rate in the exponential model in (b.1). (b.3) Growth and death rates are not simultaneously identifiable from these data, as evidenced by the strong positive posterior correlation inferred via Bayesian inference. Fig. S4-S6 show how the prior on death rates supports inferring plausible solutions for growth and death rates. (c) Logistic growth model: Growth of *E*. *huxleyi* (data in black dots) modelled (in grey lines) without and with explicit cell death information in (c.1) and (c.2-c.3), respectively. (d) Resource-explicit Monod growth: Blue lines are model solutions with parameters randomly drawn from the posterior distribution. Here, nutrients remain latent (unobserved) yet predicted via Bayesian inference in (d.3). See Supplementary Information Figs S3–S9 for details.

Many processes in microbial ecology can be modelled via birth-death processes. In a birth-death process, individuals in a population (e.g. molecules, cells or organisms) increase in number through ‘birth’ and decrease through ‘death’. Birth and death rates may depend on population densities, traits and environmental factors (see Fig. 1a). In this primer, we build examples from published work on algal systems, with increasing model complexity, as a gateway to inference for systems with greater diversity. We start with an exponential growth model, then move to a logistic growth equation and finally study the resource explicit Monod model (see Fig. 1b-d for schematics).

### Resolving parameter identifiability with informative priors: a case study with exponential growth models

**Fig. 2.**
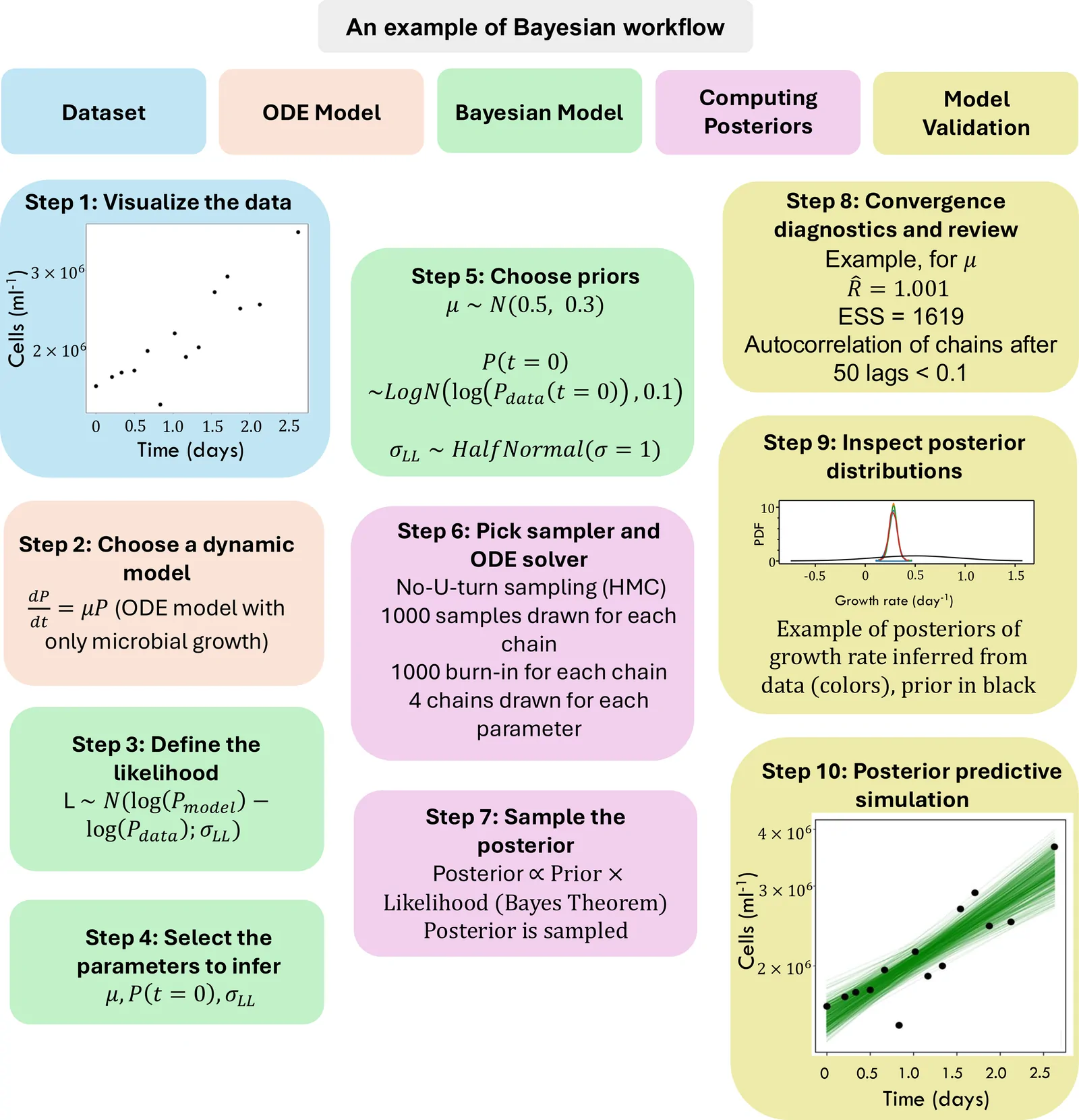
Ten-step Bayesian inference workflow for microbial systems. Demonstrated through an example of the exponential growth model on algal growth data, we show 10 steps on how to perform Bayesian inference with ODEs, which is further detailed in the Supplementary Information section S4.

We begin by analysing the growth behaviour of *P. globosa*, an alga that drives bloom formation across aquatic ecosystems worldwide ^1^. We fit an exponential growth model to experimentally recorded dynamics of this alga growing for 3 days *in vitro* (Fig. 1b.1). Because this is a well-studied system, strong priors on growth rates are available from the literature. Following the ten steps of Bayesian inference (Fig. 2), we fitted an exponential growth model to the growing population. This yielded an effective growth rate of 0.3±0.1 day^−1^. Bayesian approaches provide an advantage here by integrating prior information (in black) with the likelihood function constructed from data (in blue) to return full posterior distributions (red shade) that quantify uncertainty, rather than point estimates alone (Fig. 1b.2 and Supplementary Information Fig. S1).

Fitting an explicit death rate to these data only leads to non-identifiability of both growth and death rates, as the model is underdetermined. An underdetermined model has more parameters than required to uniquely specify its dynamics – hence, not all the parameters can be inferred from the data alone (Fig. 1b.3). What can be inferred from the data alone is an effective growth rate. However, if prior information on death rates is available, this can be incorporated. Informative priors on death rates shift the inferred growth rate upward to 0.4–0.8 day^−1^ (Fig. S2) ; fitting statistics in Supplementary Information Figs S4 and Tables S1 and S2)

To demonstrate the role of priors, we repeated the inference with different prior uncertainty on death rate. As prior uncertainty decreased, posterior uncertainty around growth rates also narrowed (Supplementary Information Fig. S5), illustrating how informative priors resolve non-identifiability even in the absence of direct death measurements. However, informative priors are only beneficial when they are ecologically grounded, as misinformed priors can bias the posterior. To demonstrate this, we performed multiple inferences with different fixed values of prior mean death rates and showed that the estimated growth rate might depend on the chosen prior, while the estimated net growth rate remains unchanged (Supplementary Information Fig. S6 and Section S6).

### Sequentially resolving parameter identifiability with additional data: a case study with logistic models

We utilized a logistic growth model to fit batch culture data of *E. huxleyi* ^2^, an alga central to marine food webs (Fig. 1c1). Using uniform priors on rates and initial densities, we inferred a growth rate of 0.5–0.7 day^−1^ and a carrying capacity of 3.1–3.4×10^6^ cells ml^−1^ (fitting statistics in Supplementary Information Fig. S7 and Tables S1-S2). The carrying capacity places an upper bound on cell density without distinguishing between static equilibrium (cells stop growing) and dynamic equilibrium (cell death balanced by new growth). Additional measurements of dead cell density using a Sytox-based stain revealed that the population stabilizes dynamically (Fig. 1c2-3). We, therefore, modified the logistic model to include an explicit constant, per-capita death term alongside the carrying capacity and performed another Bayesian inference, where priors of growth rate, carrying capacity and initial population were informed by the previous model run. With the addition of observations of dead cells, both growth rates and death rates are identifiable parameters. We inferred a death rate of 0.025–0.050 day^−1^, which is less than 10% of the net growth rate. Using additional dead cell measurements, we were able to further constrain growth rate estimates to 0.53–0.65 day^−1^. Furthermore, the data model integration revealed meaningful biology – cellular death near carrying capacity rather than just stalled growth (fitting statistics in Supplementary Information S8 and Tables S1-S2). This case demonstrates how integrating additional data with an existing Bayesian model improved parameter certainty and interpretation.

### Extension to more complex models: Monod growth model with explicit resources

We next extended the logistic equation to a resource-explicit Monod growth model with explicit mortality using the same *E. huxleyi* dataset from the previous example. In the Monod model, the growth rate is no longer assumed to be constant, but decreases with decreasing nutrient concentration, *N*, which is not directly measured in the experiment (it is a ‘latent’ variable). Leveraging known features of the growth medium, we set the prior for the initial nutrient concentration *N*_0_ centred around 880 mmol m^−1^ [3]. We informed priors of the death rate, initial live and dead cell density from the posteriors of the previous example. In the exponential growth phase (0–9 days), observed rates suggest a maximum growth rate *µ*_max_ of 0.4–0.7 day^−1^, The half-saturation constant, *K_S_* denotes the nutrient concentration at which the growth rate is half of the maximum and was not directly reported. So we set a uniform prior for *K_S_* spanning 0.05–0.4 *µ*M for nitrate across a broad range of cell sizes. The molar quota, *Q_N_*_,_ describes the average amount of nitrogen stored in a single *E. huxleyi* cell. We assumed a uniform prior from 2×10^−10^ to 3×10^−9^ mmol N cell^−1^ (priors and their sources are listed in Supplementary Information Table S1).

Posterior sampling of the full parameter set revealed strong correlations (Pearson correlation coefficient>0.9) between *N*_0_ and *Q_N_*_,_ indicating that the system is underdetermined. To address this, we computed the correlation of *N*_0_ and *Q_N_* from the posterior and reparameterized *Q_N_* in terms of *N*_0_ (Supplementary Information Fig. S9a and Methods S7). In addition, model dynamics were relatively insensitive to *K_S_* in this regime. Post-inference, the reparameterized model captures the observed saturation of algal density between 5–10 days as a consequence of both resource depletion and explicit mortality (Fig. 1d1-3). This is a key distinction from the logistic model, which does not explicitly capture resource-driven growth. Posterior estimates indicate a maximum growth rate *µ*_max_ of 0.46±0.02 day*^−^*^1^, with death rate *δ* less than 10% of *µ*_max_. By inverse parameter modelling, we inferred nutrient quota, *Q_N_*_,_ around (3.16±0.37) *×* 10*^−^*^10^ mmol N cell*^−^*^1^ without any explicit stoichiometric measurements. The Bayesian estimate of the nutrient quota is consistent with estimates of *Q_N_* computed as the ratio of *N*_0_ (*∼* 880×10*^−^*^6^^5^ mmol ml^−1^) to the difference between final and initial cell densities (*∼* 3×10^6^ cells ml^−1^) as 2.9×10*^−^*^10^ mmol N cell*^−^*^1^. Although not directly observed, we predicted that resources are depleted around 5–7 days (fitting details in Supplementary Information Fig. S9 and Table S2). In summary, this case study highlights (i) the inferring capability of Bayesian inference even with underdetermined models via model reparameterization and (ii) the predictive power of Bayesian inference over unobserved variables that influence the observed data.

## How to do Bayesian inference: a ten-step workflow

The following ten-step Bayesian workflow is a minimal, reproducible sequence we recommend using when inferring microbial traits from time series data using ODE model representations of population dynamics, as illustrated in the three case studies presented here. A visual summary of the ten-step workflow is provided in Fig. 2 and a more detailed explanation of these concepts is provided in the Supplementary Information Section S4 and the reading list [4, 8–10].

1. **Visualize the data**. Population time-series may include absolute counts (e.g. cells ml*^−^*^1^) or relative abundances (e.g. sequencing reads), often with replicates and substantial noise. Plot linear or log-scale trajectories, replicates and error bars, where applicable. Assess orders of magnitude, variability and trends before modelling. Detect outliers or anomalies and determine whether to include them in the Bayesian analysis.
2. **Choose a dynamic model**. Select an ODE with potential to represent the observed dynamics. Parameters should map directly to biological traits. This often becomes an iterative process where the model is updated in light of new observations and evidence. Note that there are quantitative approaches for discriminating between different dynamic models, but these are beyond the scope of this primer [but see Chapter 9 of [4].
3. **Define the likelihood**. Specify the data error model. It is most common to assume cell-count data are normally distributed after log-transformation. For multiple variables or replicates, take the product across log-transformed observations and model at each time point and across all time points and replicates. We suggest keeping the likelihood scale consistent with biological interpretation (e.g. compare log-transformed data when densities span orders of magnitude).
4. **Select parameters to infer**. Decide which biological traits and initial conditions are targets of inference and potentially hold constant those values where the model is relatively insensitive to its change.
5. **Choose priors**. Incorporate physical and biological parameter bounds into prior distributions of parameters. Common choices include informative Gaussians when literature exists, log-normal for parameters that scale multiple orders of magnitude, strict positivity for standard deviations and uniform when prior information is unavailable. If priors are poorly chosen, they might negatively affect the inference outcome. Therefore, simulate dynamics to verify that priors produce biologically plausible trajectories.
6. **Pick sampler and ODE solver**. This is critical, as numerical instabilities in the ODE solver are common for many systems. We recommend using gradient-based samplers (NUTS/HMC) when possible. When numerical instabilities arise, as is common for stiff ODEs, use Metropolis samplers with robust numerical integrators (e.g. solve_ivp in Python).
7. **Sample the posteriors**. Run multiple parameter chains, discard the ‘warm-up’ phase and obtain sufficient independent draws.
8. **Convergence diagnostics and review**. Check Gelman-Rubin statistics, *R*ˆ *≈* 1, effective sample size (ESS) and autocorrelation to ensure proper mixing (an explanation of these statistics is provided in Supplementary Information Section S4.8). Aim for at least 10^3^ effective parameter samples for key summaries. Inspect trace plots and pairwise plots for multimodality. Reparameterize the model if posteriors show strong correlations or review priors if diagnostics fail.
9. **Inspect posterior distributions**. Compare posterior marginals with their corresponding priors to see how much the data updated prior beliefs. Visualize credible intervals, overlaps and parameter correlations to assess sensitivity, uncertainty and identifiability.
10. **Posterior predictive simulation and visualization**. Solve the ODE model with many random draws from the posterior distributions to yield credible bands for the population dynamics (e.g. green lines in Fig. 1b.1).

It might be necessary to tune sampler hyperparameters (step size, adapt length, target acceptance, etc.) iteratively. Reporting priors, solver settings, number of chains, warm-up draws, total draws, R ˆ and ESS as supplementary information are part of best practices. Furthermore, providing the inference code and prior specification is critical to ensuring reproducibility.

## Summary and outlook

This primer has shown how Bayesian inference can be used to link mechanistic mathematical models with microbial population time-series data to extract biologically meaningful traits. Our case studies highlight three recurring themes: (i) prior information can reduce issues related to challenges of identifiability, (ii) additional datasets can provide deeper insight into parameters and (iii) reparameterization guided by posterior covariances can be a practical route to simplify models without losing biological interpretation. Together, these strategies move trait estimation beyond prior-agnostic ‘curve-fitting’ toward uncertainty-aware, mechanistically grounded inference. Microbial traits, such as growth rates, death rates, and resource use efficiencies underlie phytoplankton bloom dynamics, microbial community interactions and biogeochemical cycling. Trait inference directly informs our ability to predict microbial responses to environmental change, to identify limits on productivity and to design or control microbial communities in applied settings. By explicitly quantifying uncertainty, Bayesian methods reveal not only what we know but also where data or models are insufficient, so informing reproducible, predictive microbial ecology. Bayesian inference comes with its own set of challenges and results depend on subjective biological assumptions. For example, in case study 1, *P. globosa* death rates were taken from cultures that were not in exponential growth and, therefore, may be in a different physiological state. Furthermore, our logistic and Monod models assume a closed system and do not account for nutrients lost through exudation. The Bayesian framework can account for these limitations as new data on cell death and exudation emerge. On the computational side, there are numerous challenges and decisions to be made in fitting. Samplers can be computationally intensive, sensitive to ODE solver accuracy and dependent on prior choices, especially when data are sparse. Yet modern probabilistic programming frameworks, modern MCMC samplers and diagnostic tools make these challenges increasingly tractable. In closing, Bayesian inference provides a flexible and principled framework for connecting ecological theory, mechanistic models and microbial data. By embracing uncertainty and identifiability, microbial ecologists can extract more robust and biologically meaningful trait estimates. We hope that this primer, with worked examples, template code and cross-language support, lowers the barrier to entry and helps integrate Bayesian thinking into the experimental and theoretical toolkit of microbial ecology.

## Supplementary material

10.1099/mic.0.001739 Supplementary Material 1.

## Footnotes

1. Data from: A-C Baudoux and Corina PD Brussaard. ‘Characterization of different viruses infecting the marine harmful algal bloom species Phaeocystis globosa’. In: Virology 341.1 (2005), pp. 80–90.

2. Data from: Noa Barak-Gavish et al. ‘Bacterial virulence against an oceanic bloom-forming phytoplankton is mediated by algal DMSP’. In: Science advances 4.10 (2018), eaau5716.

## Abbreviations

ESS: effective sample size
MCMC: Markov Chain Monte Carlo
ODE: ordinary differential equation
