# Supplementary material for: Microbial Primer: Bayesian learning of traits from microbial time series data
Source: Microbiology (Reading). 2026 Aug 10;172(8):001739. doi: 10.1099/mic.0.001739 (PMC13456637; doi:10.1099/mic.0.001739)
Supplement: Supplementary file 1 [file mic-172-01739-s001.pdf]

Supplementary Information

for

Microbial Primer: Bayesian learning of microbial traits from population time-series data

Raunak Dey[1,*], Robert Beach[2], Kennedi M. Hambrick[2], Ioannis Sgouralis[3], Paul Frémont[4], David Demory[5], Eric Carr[2], Stephen J. Beckett[4,6], Joshua S. Weitz[4,6], David Talmy[2,*]

[1] Department of Physics, University of Maryland, College Park, MD, USA
[2] Department of Microbiology, University of Tennessee Knoxville, Knoxville, TN, USA
[3] Department of Mathematics, University of Tennessee Knoxville, Knoxville, TN, USA
[4] Department of Biology, University of Maryland, College Park, MD, USA
[5] Sorbonne Université, CNRS, UMR8176 Laboratoire de Biodiversité et Biotechnologies Microbiennes (LBBM), Observatoire Océanologique, F-66650 Banyuls-sur-Mer, France
[6] University of Maryland Institute for Health Computing, North Bethesda, MD, USA

[*] Correspondence: rdey@umd.edu; dtalmy@utk.edu

S1 Glossary of Bayesian Terminology

Autocorrelation Plots Plots showing correlation between samples separated by different lag times; used to assess dependence between draws. High correlation between draws indicates inefficient exploration of parameter space.

Burn-in The initial portion of an MCMC chain that is discarded because the sampler has not yet converged.

Chain A sequence of parameter values produced by an MCMC algorithm to explore the posterior distribution.

Corner plot One and two dimensional projections of the posterior probability distribution for the model parameters, typically shown for multiple parameter combinations, that allow identification of correlations and/or multimodality in the posterior.

Convergence The point at which an iterative sampling procedure stabilizes and approximates the target distribution.

Credible Interval A range of parameter values containing a specified proportion (e.g., 95%) of the posterior distribution.

Draw A retained posterior sample recorded at each iteration after burn-in/tuning.

Gelman-Rubin statistics A statistical test for MCMC chain convergence that compares the between-chain variance to within-chain variance for the same parameter.

Hierarchical Bayesian A Bayesian model with parameters organized in multiple levels, typically with group-level hyper-priors (higher-level) governing individual-level priors (lower-level).

HMC Hamiltonian Monte Carlo. Uses concepts from Hamiltonian dynamics to generate efficient proposals and explore parameter space more effectively.

Identifiability The property that model parameters can be uniquely determined from the available data.

Informative vs. Non-informative Prior Informative priors incorporate domain knowledge, often using distributions such as a Normal, Gamma, or Beta distribution. Non-informative priors contain minimal assumptions (e.g., Uniform), allowing the data to dominate the inference.

Leapfrog dynamics A particle dynamics where the positions are discretely computed at full time steps and momentum is computed at half time steps to ensure energy conservation and time-reversal.

Likelihood A measure of how well the model-generated data explain the observed data.

Marginal A distribution obtained by integrating out all other variables, leaving the distribution of only a subset of variables.

MCMC Markov Chain Monte Carlo. A sampling framework where: (1) "Markov chain" indicates that each step depends only on the previous step; (2) "Monte Carlo" indicates the use of random sampling.

Mixing A measure of how well an MCMC chain explores the parameter space.

Multimodality A situation where a posterior distribution has more than one mode.

Pairwise Posterior Plot A visualization of joint posterior relationships between pairs of parameters (scatter or contour).

PDF or Probability Distribution Function The probability of a continuous-valued parameter falling within a particular range of values. Since it is probability density rather than probability it can be greater than one – the PDF integrated over the whole range of parameters gives unity.

Posterior A probability distribution of model parameters obtained by combining the prior with the likelihood.

Posterior Predictive Check A procedure to assess whether data simulated from the posterior distribution resemble the observed data.

Prior A statistical distribution representing prior knowledge about parameters. Priors may be informative or non-informative.

Prior Predictive Check A procedure to assess whether the chosen priors generate plausible simulated data before observing real data.

PyMC An open-source Python package for Bayesian modeling and inference.

Sampler An algorithm that generates samples from a probability distribution, especially when direct sampling is difficult.

Sensitivity The change in the model trajectories with a change in the values of the parameters.

Solver A numerical algorithm for approximating solutions to mathematical problems such as ordinary differential equations (ODEs).

Stiff ODE An ODE where nearby solutions are fast varying whereas the sought-after solution is slow varying, leading to numerical errors in integration unless very small time-steps are chosen.

Trace plots Plots showing sampled parameter values across iterations to assess convergence and mixing.

Tune The adaptive warm-up period prior to sampling, used to adjust the sampler for improved efficiency.

Turing An open-source Julia package for Bayesian modeling and inference.

S2 Dataset and case studies

All the datasets used in this work were extracted from published work using a plot-digitizer toolkit [1]. For the algae growth and growth-death models in Section 1, we used the example of *Phaeocystis globosa* grown in ESF2 medium, a 1:1 mixture of f/2 medium and enriched artificial seawater with the addition of Tris–HCl and Na_2SeO_3 [2]. For the other two models, we used *Emiliania huxleyi* as our model organism, due to its ecological significance in global biogeochemical cycles [3]. We analyzed the growth and death dynamics of *E. huxleyi* monocultures growing in batch culture conditions for 17 hours. For that, we extracted the data from Fig. 1 B and C of Ref. [4]. We provide three case studies with these datasets and the ODE models, described below.

S3 Cross-platform tools and tutorials

Alongside this primer, we provide online resources of cross-language and multi-package programming support for Bayesian inference of microbial traits. Our tutorials, archived at `https://b2-bayesian-for-biology.github.io/MCMCwithODEs_primer/`, guide installation, code templates, methodology, and further details on the case studies. The examples in this article are analyzed with PyMC (Python), whereas the online tutorials provide support for both PyMC (Python) and Turing (Julia) [5, 6, 7, 8, 9, 10, 11]. Beyond the reading list, interested readers might also appreciate additional examples of fitting biological ODEs to experimental data from microbial and phage systems [12, 13, 14, 15, 16, 17].

All analyses were performed using Python 3.12 with PyMC v5.25.1, PyTensor v2.31.7, ArviZ v0.22.0, NumPy v2.2.5, SciPy v1.16.2, and Julia v1.11.0 with Turing.jl v0.40.2, DifferentialEquations.jl v7.16.1, SciMLSensitivity.jl v7.90.0, Distributions.jl v0.25.120, and MCMCChains.jl v7.2.0. Complete environment specifications and reproducible code are available at the project repository via GitHub: `https://github.com/B2-Bayesian-for-Biology/MCMCwithODEs_primer`

S4 Tutorial for 10 steps of Bayesian Inference

We recommend using the following steps for a Bayesian workflow as visualized in Fig. 2.

S4.1 Visualize the data

In each case, begin by visualizing the dataset to develop qualitative understanding of the behavior of each population over time. When population densities span several orders of magnitude they are best visualized on logarithmic Y-axes.

S4.2 Choose a dynamic model

After qualitatively observing the change in population density we choose a quantitative dynamic model for our system.

Exponential growth and death: We started with a simple example where the population rate of change is proportional to the population density itself. The exponential growth process is given by,

$$\frac{dP}{dt} = \mu P \tag{3}$$

where, P is the cell population (ml^{-1}) and μ is the growth rate (day^{-1}). Next, we also include a death term, where the death is also proportional to the current population density, as a rate δ, and the ODE is given by,

$$\frac{dP}{dt} = \mu P - \delta P \tag{4}$$

Logistic equation: Visualizing the slowdown of growth of *E. huxleyi* in the second case, we introduced a Logistic growth term that phenomenologically captures the steady state behavior. Here, growth is still proportional to the current population density P'; however, there is a bottom-up or top-down control that hinders population growth as the density approaches a value of K, known as the carrying capacity. The ODE may be written

$$\frac{dP'}{dt} = rP'\left(1 - \frac{P'}{K}\right) \tag{5}$$

Here, we do not distinguish between living and dead cells. Subsequently, after visualizing the dead cell density, D, through our experiments, we added a death process where the number of dead cells increases at a rate δ, proportional to the present live population density, P. So total cells is $P + D$. The ODE is given by,

$$\frac{dP}{dt} = rP\left(1 - \frac{P}{K}\right) - \delta P \tag{6}$$

$$\frac{dD}{dt} = \delta P \tag{7}$$

Explicit Monod growth rate: In this case, we explored a resource explicit model with Monod growth. In the Monod growth model, the growth rate varies with the nutrient concentration N. Cells grow at maximum rate μ_{max}, but the rate of growth drops to half of the maximum at a nutrient concentration of K_S. The fixed cellular nutrient quota Q_N is expressed in molar units, and sets the mols of nutrient consumed with each new cell produced. As in previous cases, the cells die at a rate δ proportional to the living cell density P. The selected units for P were ml^{-1}, for N were mmol N m^{-3} and for Q_N were mmol N cell^{-1}. To ensure unit consistency, P is multiplied by 10^6 ml m^{-3} in the N ODE.

$$\frac{dN}{dt} = -\frac{Q_N \mu_{max} N}{N + K_S}(P \times 10^6 \text{ ml m}^{-3}) \tag{8}$$

$$\frac{dP}{dt} = \frac{\mu_{max} N}{N + K_S} P - \delta P \tag{9}$$

$$\frac{dD}{dt} = \delta P \tag{10}$$

S4.3 Define the likelihood function

A likelihood function is defined as the probability of observing the data $\mathcal{D}$ given the parameter set θ of our model, which is often written as $\mathcal{L}(\mathcal{D}|\theta)$. In the case where the ODE model captures observed dynamics, the likelihood has sharp peaks, compared to the case with more pronounced model-data deviation and a relatively diffuse likelihood distribution.

A common example is a Gaussian likelihood function, where the variation of the model from the observed data is modeled by a normal distribution, with a mean around the observed data and the variance tuned to how far the model deviates. Since there can be multiple data points in each variable (alongside multiple replicates and multiple variables), often the likelihood function is defined by the product of many normal distributions around each point. In the case where all the points are treated with equal weight, the log of the likelihood function only depends on the sum of squared errors between model and data points, and the variance of the Normal distribution. As is often the case with population time series, the data in our three case studies all varied over many orders of magnitude; therefore, we compared the log-transformed model and data via a Gaussian likelihood that treats all data points with equal weights. See Table S1 for likelihood functions used. In constructing the likelihood function, we also note that in some cases, variables might be latent (unobserved), and the likelihood function is constructed only with the available data.

S4.4 Select parameters to fit

In the first two cases, we fitted all the parameters of the corresponding dynamical system, including the initial conditions and the variance of the likelihood function. In the last case, after fitting all the parameters, where we found high posterior covariance, we chose to reparameterize our model by defining one parameter via the other. In that case, we chose to fit the independent parameter only, as the dependent parameter is defined internally.

S4.5 Choose priors

In our examples, priors were informed by literature. Although different probability distributions can be chosen, in our examples, we primarily assumed Normal distributions. When no prior information was available for a given prior, we chose a Uniform distribution, which assigns equal probability to all plausible parameter values (non-informative prior). When prior information was available, we chose Gaussian priors with their means and variances informed by the literature. For parameters spanning orders of magnitude, like population densities, Log-Normal priors were assumed.

For the prior standard deviation of the likelihood function, a half-Normal distribution was assumed to ensure strict positivity and the model to be normally distributed around the data points. To ensure positivity in some of the rates, such as growth and death rates, we also implemented truncations (an upper and/or a lower bound) in these distributions. When the likelihood function has small variance, indicating that the observations are known with high certainty, priors tend to have less influence in determining the posterior distribution.

Since prior assumptions can be subjective, to ensure reproducibility, it is important to properly specify them. In addition to the datasets, the priors for fitting *P. globosa* and *E. huxleyi* were also informed by the literature. See Table S1 for the list of priors and references. Please refer to Supplementary Information Table S1 for the list of priors used in this primer.

S4.6 Pick sampler and ODE solver

Numerous algorithms have been developed to approximate posteriors via Bayesian inference, ranging from Metropolis and Metropolis-Hastings algorithms to Hamiltonian Monte Carlo, Approximate Bayesian Computing, Variational Inference, etc., with each having advantages and disadvantages. Here, for the first two cases, we used No-U-Turn-Sampling (NUTS) to sample the joint posterior distribution. This algorithm adaptively sets the number of steps in the Hamiltonian simulation, eliminating the need for manual tuning [18, 19]. NUTS needs to access or compute the gradient of the variables with respect to the parameters (Jacobian), which is either provided or computed internally, and might become challenging for stiff ODE systems. We used `DifferentialEquation` from the PyMC package as the differential equation solver with NUTS. For case 3, the differential equations were stiff for some parameter sets, leading to difficulty computing the gradient with NUTS. So we reverted to a simple Metropolis algorithm, with `solve_ivp` from the `SciPy` package as a solver.

Hyperparameters of the samplers: The hyperparameters of these samplers are not part of the statistical model or the ODE model, but they govern the convergence of the sampler to the posterior, mixing efficiency, computational cost and are typically tuned automatically in the burn-in phase.

For samplers of the Metropolis family, such as in the Metropolis-Hastings algorithm, the main hyperparameter is the covariance matrix of the proposal distribution that controls the step size of the proposal from the current position of the sampler. A larger step size encourages wider exploration, but risks higher rejection rates; whereas, a smaller step size leads to higher acceptance rates but inefficient exploration of the posterior. Modern samplers can 'adaptively' tune the covariance matrix often by optimizing for the acceptance rates.

In samplers of the HMC family, such as NUTS, the key hyperparameters are integration step-size of the Hamiltonian dynamics, mass matrix that rescales the parameter space and number of 'leapfrog' steps. The step size should be

neither too small and exploring parameter space inefficiently nor too large, leading to an intolerably high rejection rate. A well-tuned mass matrix aligns the sampler with the geometry of the posterior, by rescaling and rotating the parameter space, improving efficiency and reducing autocorrelation. A well-tuned leapfrog length ensures that the sampler trajectory does not go too far from the current state thereby increasing computation or stay too close behaving like a random-walk. NUTS automatically tunes this leapfrog length by involving a 'No-U-Turn' criterion. In practice, we tuned the target acceptance rates from 0.8 to 0.95 in PyMC thereby letting the sampler adapt the rest.

S4.7 Sample the posteriors

Alongside the ODE solvers, sampler performance depends critically on tuning the sampler properties. These properties are often adjusted iteratively until satisfactory convergence is achieved. In practice, we recommend the following steps:

- **Prior predictive checks:** simulation of data from the prior before observing real data, to ensure priors lead to plausible system behavior.
- **Discarding the Burn-in (warm-up):** initial samples are discarded until the sampler adapts and begins to explore the target posterior. The required burn-in length increases with increasing model complexity and dimension.
- **Choosing the number of draws and chains:** sufficient samples and multiple chains are necessary to capture the posterior adequately and to assess convergence; we try to obtain at least 1000 independent samples for each parameter. (See ESS below).
- **Chain thinning:** optional step to reduce autocorrelation between samples, though with modern samplers like NUTS, this is less often required.
- **Tuning the acceptance rate:** acceptance rate is the fraction of proposed moves accepted; values too low or too high can indicate inefficient exploration. Increasing acceptance rates increases sampling time – the default rate is 80% in `pymc`. However, we increased the acceptance rate to 95% to ensure that convergence statistics fell within an acceptable region.

S4.8 Convergence diagnostics

Ensuring that the Markov chains have converged is critical before interpreting the posterior. Standard convergence diagnostics include:

- The **Gelman–Rubin statistic ($\hat{R}$)**, which compares within- and between-chain variance; values close to 1 indicate convergence. In our examples, we ensured $\hat{R}$ is less than 1.01 for each case.
- **Autocorrelation** analysis of each chain, to verify that successive samples are not overly dependent. In our examples, we ensured that the correlation falls below 0.1 within 50 lags, meaning the average correlation of the present sample to 50 samples behind is less than 0.1.
- **Effective sample size (ESS)**, both bulk and tail, which measures how many independent samples are effectively obtained for the posterior mean and tails, respectively. We ensured that ESS is greater than 1000 for all cases indicating sufficient mixing.

These diagnostics indicate whether the posterior has been sufficiently explored. In the case where one or more convergence tests fail, one might need to iteratively check the data, ODE framework, Bayesian model and associated hyperparameters for debugging.

S4.9 Inspect posterior distributions

Visualization plays a central role in interpreting Bayesian inference. Pairwise scatter plots of parameters ("corner plots") allow identification of correlations, multimodality, or non-Gaussian structures in the posterior. When strong correlations are observed between parameters, one may attempt a reparameterization of the model (e.g., using transformed variables) to improve sampling efficiency and interpretation. Marginal posterior distributions further summarize credible intervals for each parameter.

S4.10 Posterior predictive simulation and visualization

The posterior distributions of parameters can be propagated through the dynamical system to generate predictive trajectories. This procedure, often called *posterior predictive simulation*, produces an ensemble of model solutions that directly reflect uncertainty in the parameters. Comparing these trajectories to observed data provides a natural check of model fit, while the spread of trajectories quantifies predictive uncertainty in the dynamics.

S5 Statistical estimators for exponential growth model

We estimated the exponential growth rate parameter μ from observed trajectories using least squares, maximum likelihood, bootstrap resampling, and maximum a posteriori methods implemented in Python.

Ordinary Least Squares (OLS): estimated by fitting the exponential model to observed data using nonlinear least squares via the `scipy.optimize.curve_fit` function.

Maximum Likelihood Estimation (MLE): estimated by maximizing the Gaussian log-likelihood of the observed data given the exponential model using numerical optimization with `scipy.optimize.minimize`.

Bootstrapping: datasets were generated by sampling with replacement using `sklearn.utils.resample` and refitting the exponential model with `scipy.optimize.curve_fit`. The resulting distribution of estimates was used to compute the mean and standard deviation of μ.

Maximum A Posteriori (MAP): the negative log-posterior, defined as the sum of the negative Gaussian log-likelihood and a Normal prior term, was minimized, using `scipy.optimize.minimize`. This incorporates prior information while retaining the likelihood-based inference framework.

S6 Effect of priors on posteriors (demonstrated using case-1)

Here the underlying model is underdetermined with growth and death rates coupled, such that one has influence of the other. We studied the effect of changing priors of death rates on estimated growth rates, which are as follows.

Effect of differences in prior uncertainty on inferred growth rate: We performed five difference Bayesian inference for *Phaeocystis globosa* using Eqn. 4, where the standard deviation of the prior death rate is different in each case (ranging from 0.01 to 0.3 day^{-1}), while the prior mean death rate remains fixed.

We observed that increasing the prior uncertainty of death rate also increases the prior uncertainty around growth rates underpinning the roles of informed priors in Bayesian inference (Fig. S5).

Effect of differences in prior means on inferred growth rates: Next we performed seven more Bayesian inference for *Phaeocystis globosa* using Eqn. 4, where the mean prior death rate is different in each case (ranging from the 0.05 to 0.5 day^{-1}), but their uncertainty is fixed ($\sigma_\delta = 0.1$, ignoring truncation of the prior distribution at zero). Here, the posterior mean of the growth rate, μ, increased monotonically with the prior mean of the death rate, δ. A change of 0.45 day^{-1} in the prior mean of δ produced a $\sim$0.35 day^{-1} systematic bias in inferred μ. The sub-unity slope reflects that the prior on μ and residual information from the data moderated the effects of prior δ, but the bias was not corrected by the data alone. The mean of the net growth rate, $\mu - \delta$, is the same for each case (around 0.28 day^{-1}) as expected (Fig. S6).

S7 Reparameterization in case-3

To reduce the model complexity, while estimating parameters with the resource-explicit model, we first sampled all the parameter posteriors. Upon observing that Q_N and N_0 had a positive correlation of > 0.9, we computed the best fitting line between Q_N and N_0 using the samples of their correlated posteriors as $Q_N = 3.67 \times 10^{-13} N_0 - 2.39 \times 10^{-12}$. Post reparameterization, instead of resampling Q_N we sampled only N_0 while keeping Q_N dependent on N_0 to increase ESS and ensure convergence.

S8 Parameter correlation analysis

In this section, we analyze parameter correlations that arise naturally from the functional form of the models, and compare them with the posterior dependencies observed in our Bayesian inference (Fig. S7–S9).

Exponential growth with death. For the model in Equation 4, the solution is

$$P(t) = P_0 \exp\big((\mu - \delta)t\big).$$

Here, μ and δ appear only through their difference $(\mu - \delta)$, and are therefore not separately identifiable. This structural confounding leads to strong positive correlation between μ and δ in the posterior.

Logistic growth with death. The logistic model with an additional death term (Equation 6) can be solved as

$$P(t) = \frac{K}{1 + \left(\frac{K}{P_0} - 1\right) \exp\big((\delta - r)t\big)}.$$

From this expression, several dependencies follow. First, r and δ enter only through the difference $(r - \delta)$, and are thus strongly positively correlated. Second, P_0 and r influence the exponential pre-factor in opposite directions, leading to a negative correlation between them. While K may correlate with other parameters through saturation effects, its relationship with δ is not direct; the dominant dependencies are r–δ and P_0–r. These trends are borne out in the posterior plots (Fig. S7d, Fig. S8d).

Resource-explicit model. For more complicated models, analytical analysis is difficult, and we rely on the posterior corner plots to explore parameter covariances. We found strong positive covariance between N_0 and Q_N, and negative covariance between P_0 and $\mu_{\max}$ (Fig. S9d).

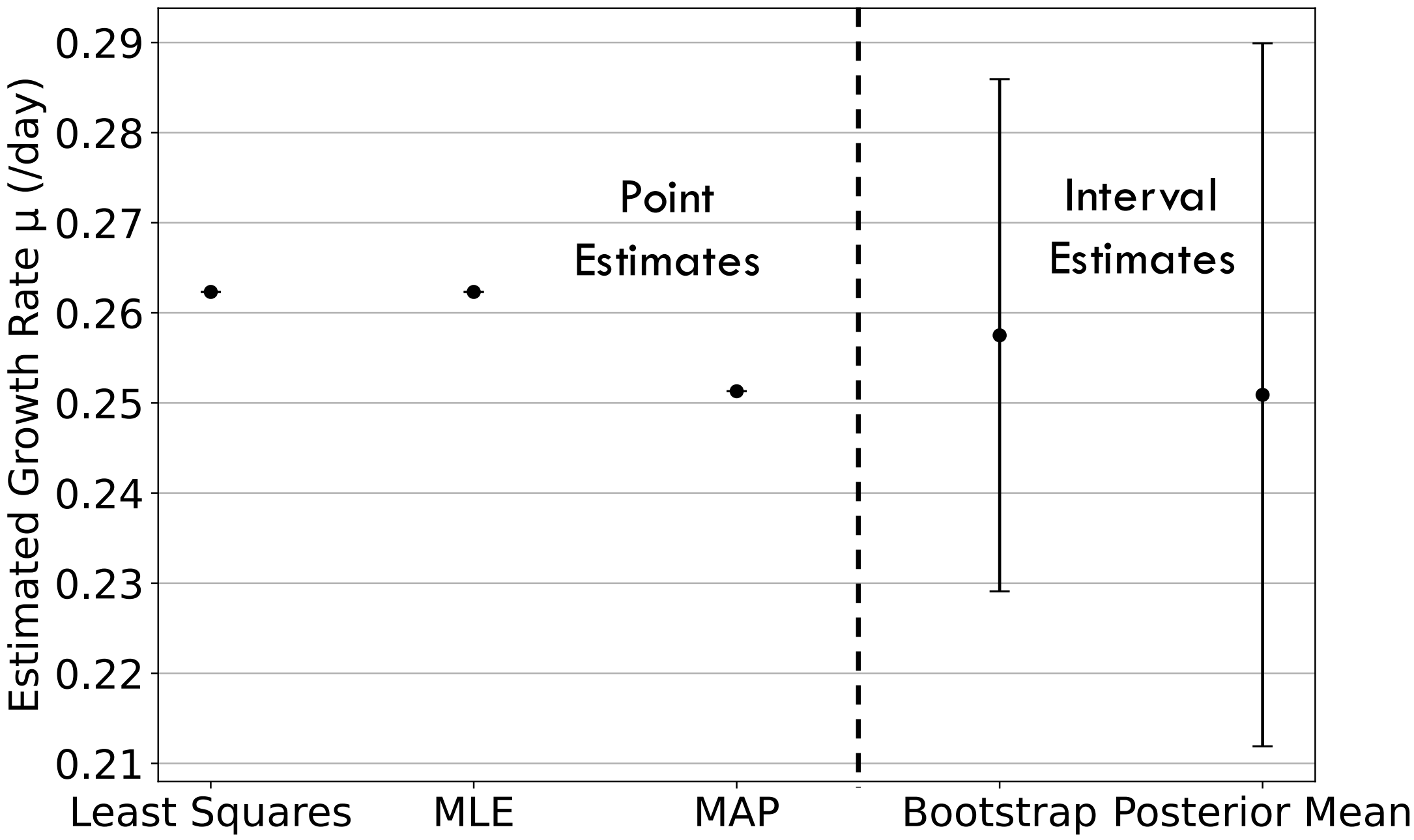

Figure S1: **Comparison of Bayesian and non-Bayesian approaches.** Growth rate of *Phaeocystis globosa* estimated from dynamics shown in Fig. 1b.1 through common non-Bayesian and Bayesian approaches (see section S5 for details). MLE - maximum likelihood estimator; MAP - maximum a posteriori

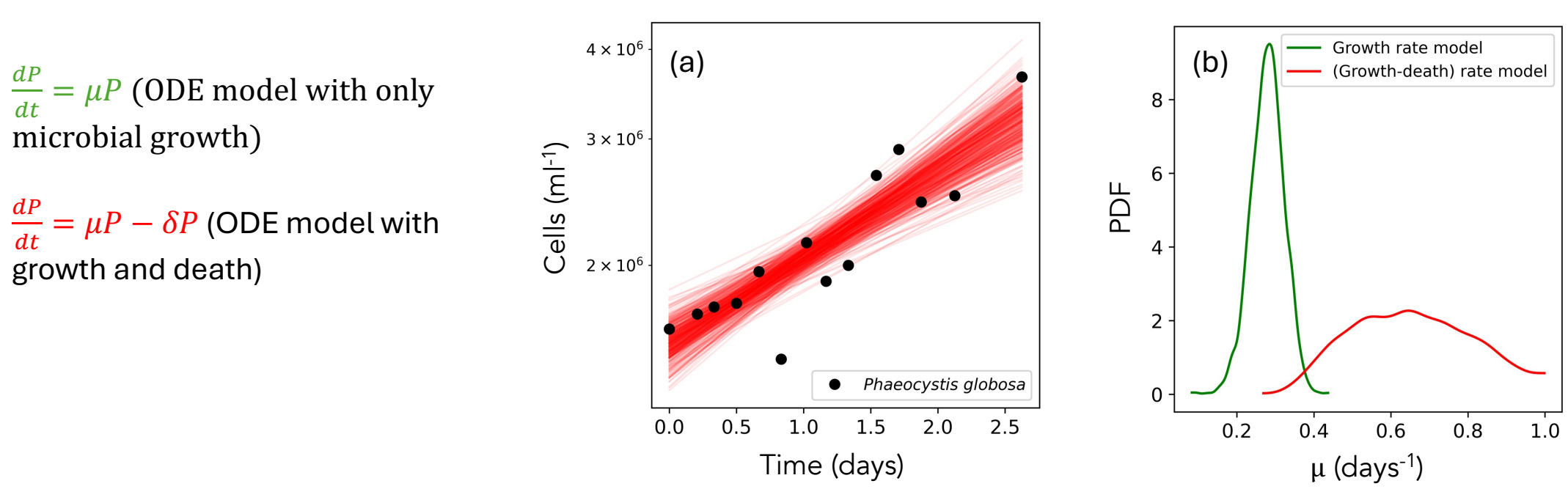

Figure S2: **Explicitly modeling microbial death.** (a) Modeling time series of *Phaeocystis globosa* from Ref. [2] via Bayesian inference and an ODE model with death. The population was modeled with both growth and death terms using Equation 3, where we chose an arbitrary Gaussian prior for death rates. (b) Explicitly modeling death leads to different estimates of growth rate.

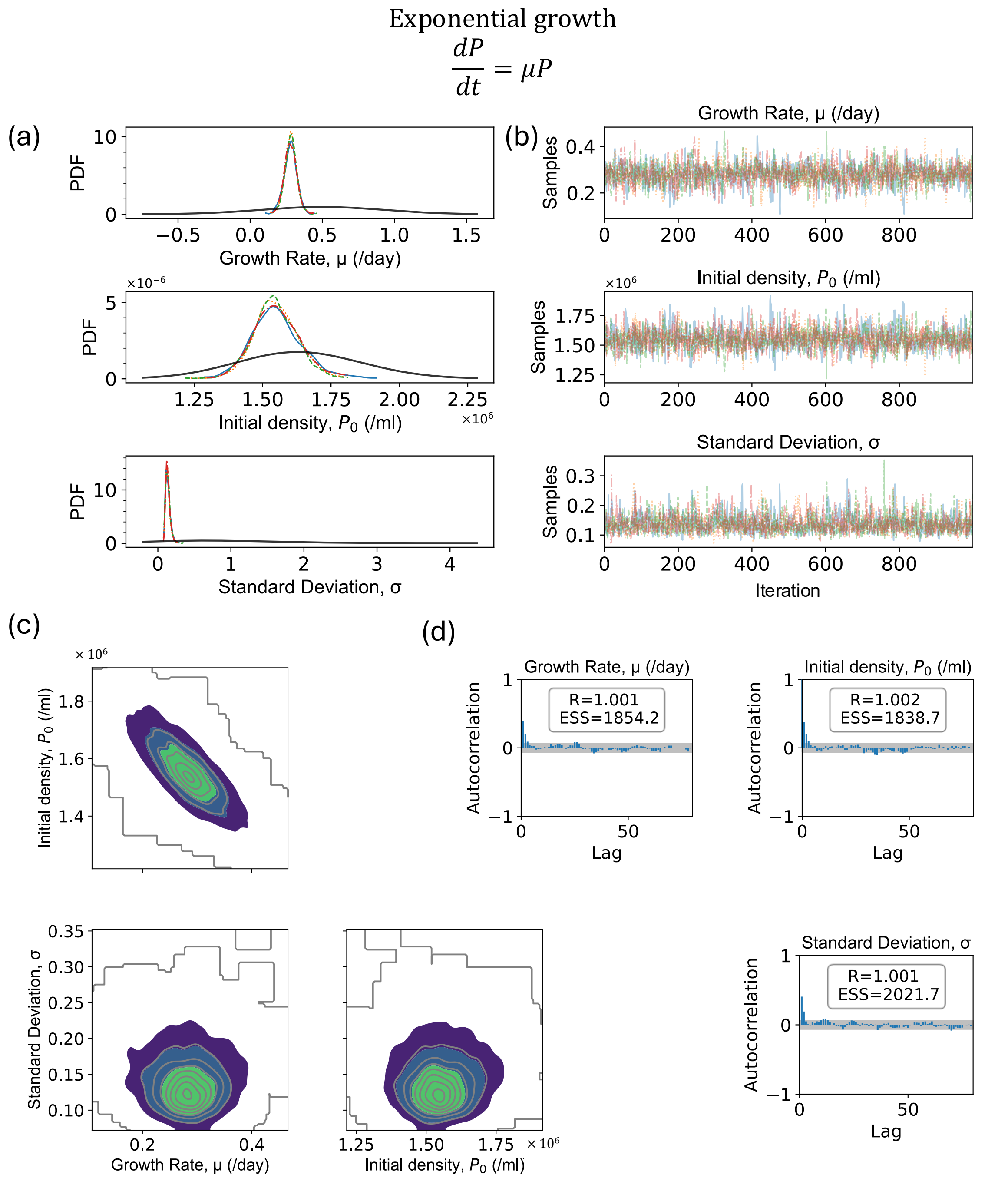

Figure S3: **Prior and Posterior statistics of fit via an Exponential model.** (a) Priors are shown in black lines, while the posterior kernel densities are plotted in colors for different chains. (b) Trace plots for independent chains for the sampled parameters. (c) Pairwise posterior plots for all the variables. Initial concentration and growth rates are found to be negatively correlated. Some degree of correlation is acceptable here, since the posteriors in (a) are clearly tightly constrained and well within the priors. (d) Convergence statistics for all the chains combined. Hyperparameters for this plot: Burn-in: 1000 points for each chain, samples drawn: 1000 points for each chain, number of chains: 4, Thinning: None, Algorithm: NUTS.

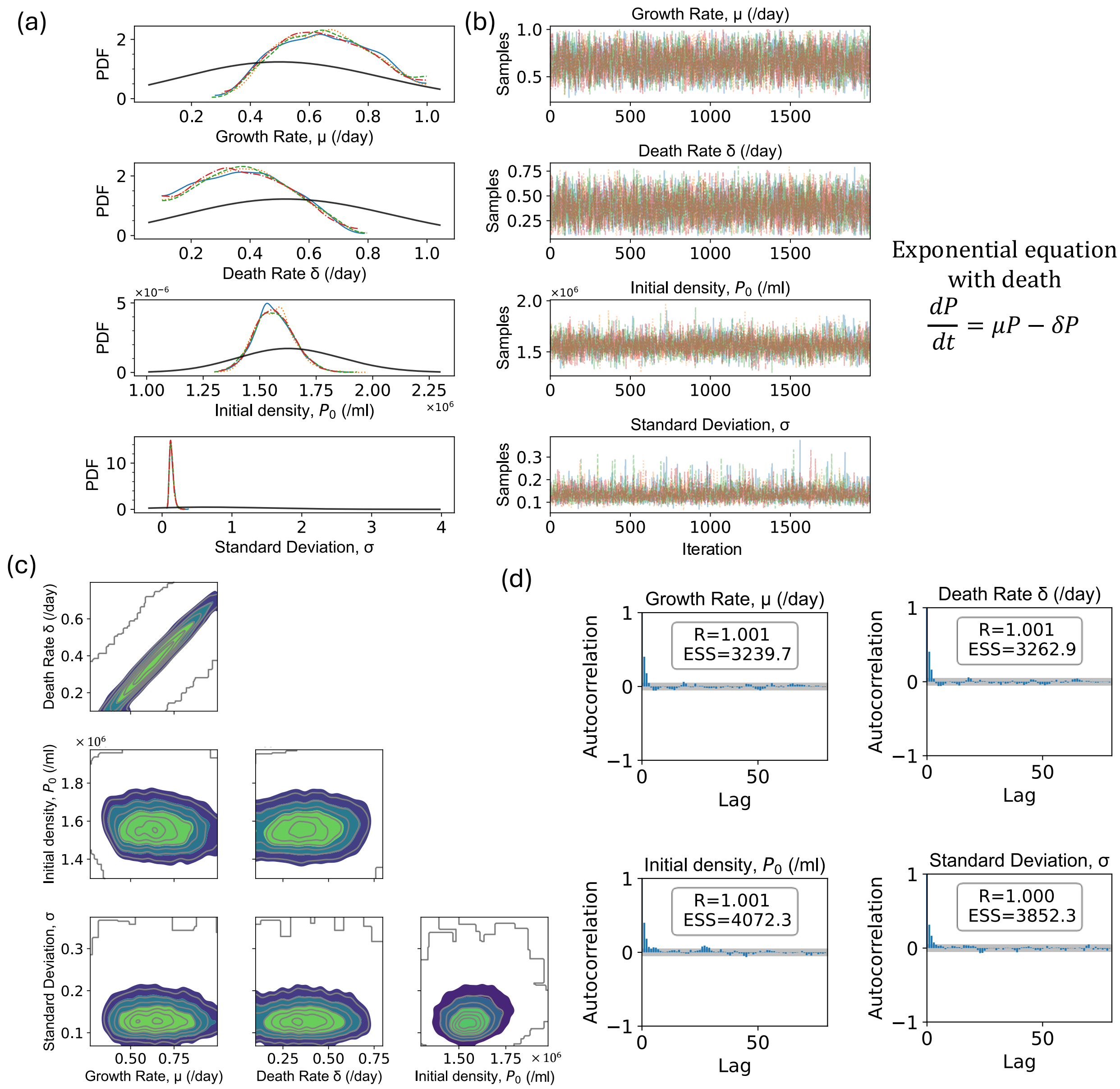

Figure S4: **Prior and Posterior statistics of fits with exponential growth and death.** (a) Priors are shown in black lines, while the posterior kernel densities are plotted in colors for different chains. (b) Trace plots for independent chains for the sampled parameters. (c) Pairwise posterior plots for all variables. Growth and death rates exhibit strong positive posterior correlation. Here, this strong correlation is a concern, since the posteriors in (a) are broad, extending beyond the priors in at least one direction. Therefore, these parameters are unidentifiable. (d) Convergence statistics for all the chains combined. Hyperparameters for this plot: Burn-in: 1000 points for each chain, samples drawn: 1000 points for each chain, number of chains: 4, Thinning: None, Algorithm: NUTS.

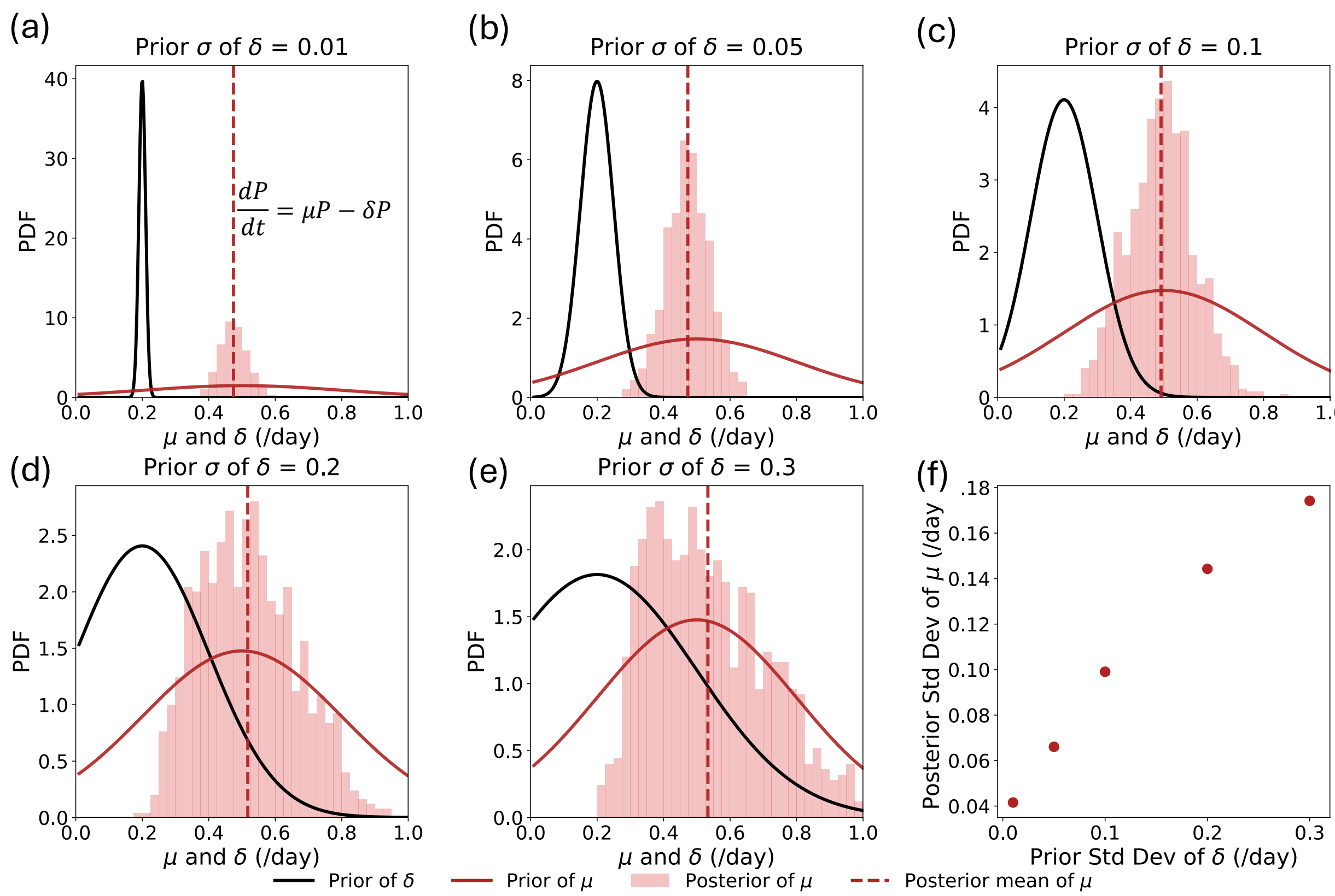

Figure S5: **Effect of death rate uncertainty on inferred growth rates.** *Phaeocystis globosa* growth rate posterior changes with changing uncertainty in the death rate prior using Eqn. 4. (a-e) Having more informative death rate priors leads to more informative growth rate posteriors. (f) Variation of the estimated μ posterior with the change in δ prior.

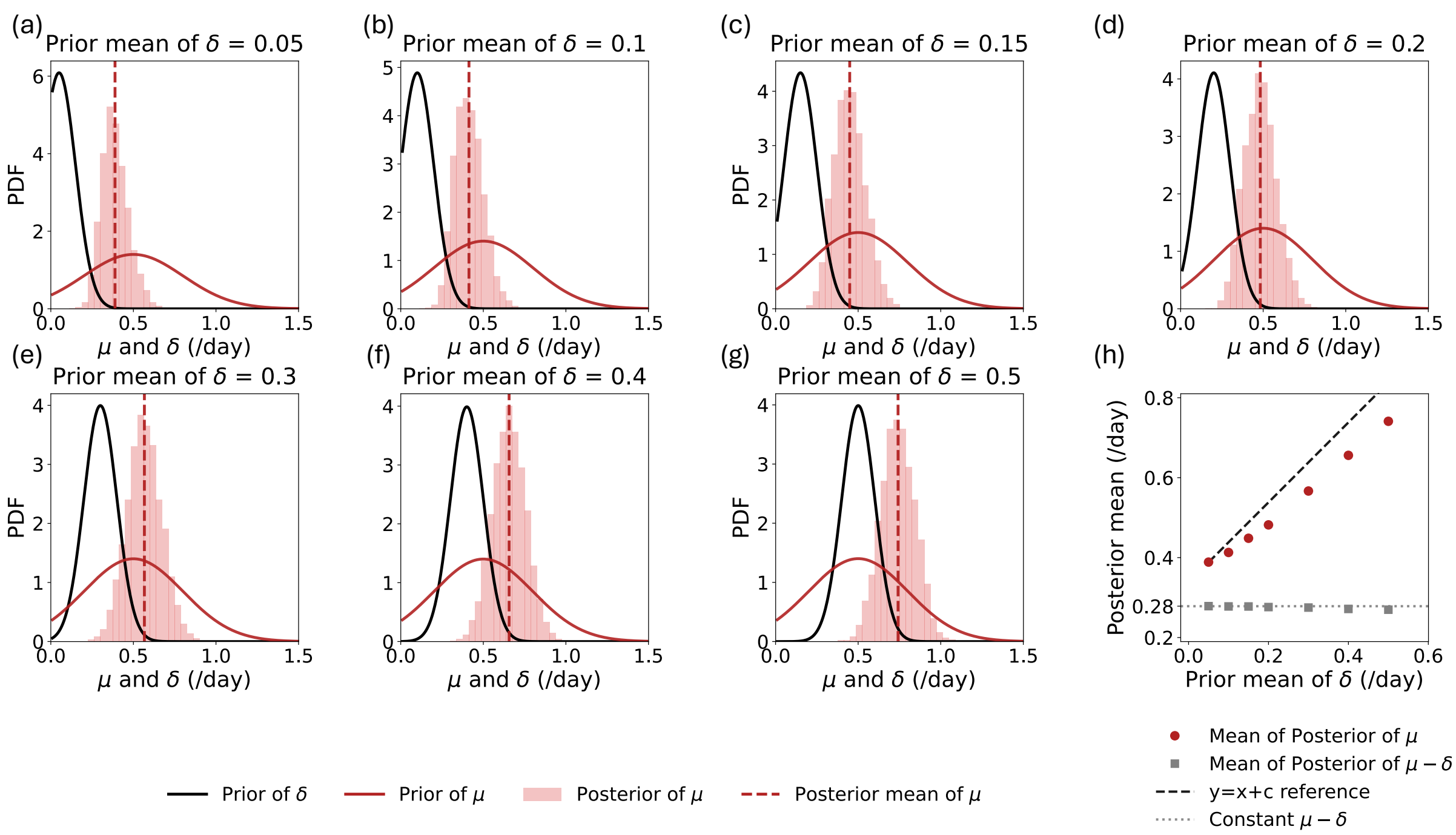

Figure S6: **Effect of differences in prior means of death rates on the inferred growth rates of *Phaeocystis globosa*.** (a–g) Posterior distributions of μ (colored histograms) for different prior means of δ (black curve), with dashed vertical lines indicating posterior mean estimates of μ. Prior of μ (red curve) remains unchanged. (h) Posterior mean of μ for corresponding prior mean of δ. Points fall below the dashed $y = x + c$ reference line, indicating the prior on μ and residual information in the data were not enough to correct the bias in estimating μ. However inferred mean of the effective growth rate, $\mu - \delta$ is $\sim$0.28 in each case.

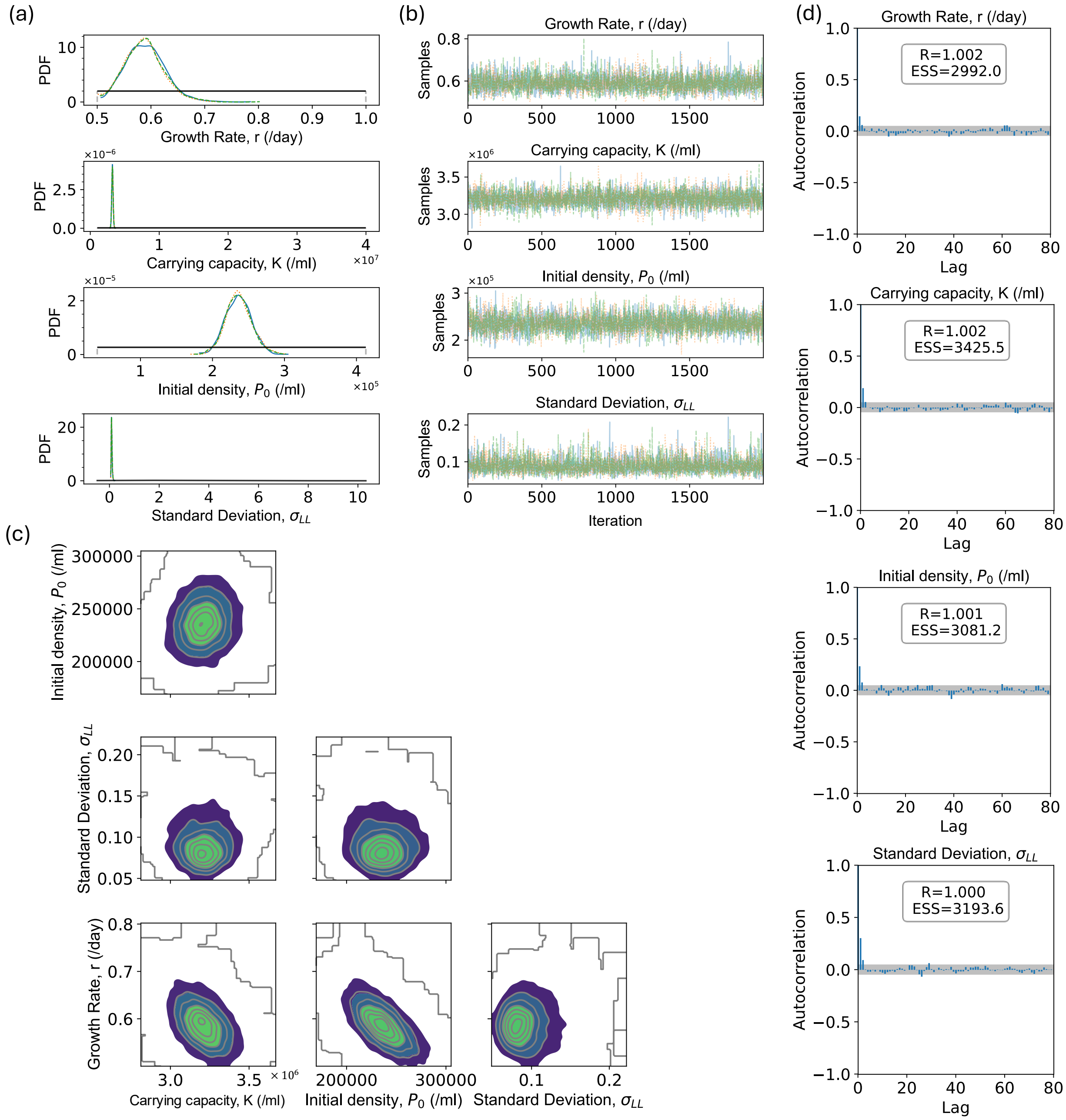

Figure S7: **Prior and Posterior statistics of fits with a logistic growth model.** (a) Priors are shown in black lines, while the posterior kernel densities are plotted in colors for 3 different chains. (b) Trace plots for 3 independent chains for the sampled parameters. (c) Pairwise posterior plots for all variables. Growth rates and initial conditions exhibit strong negative posterior correlation, similar to Fig S3. (d) Convergence statistics for all the chains combined. Hyperparameters for this plot: Burn-in: 500 points for each chain, samples drawn: 2000 points for each chain, number of chains: 3, Thinning: None, Algorithm: NUTS.

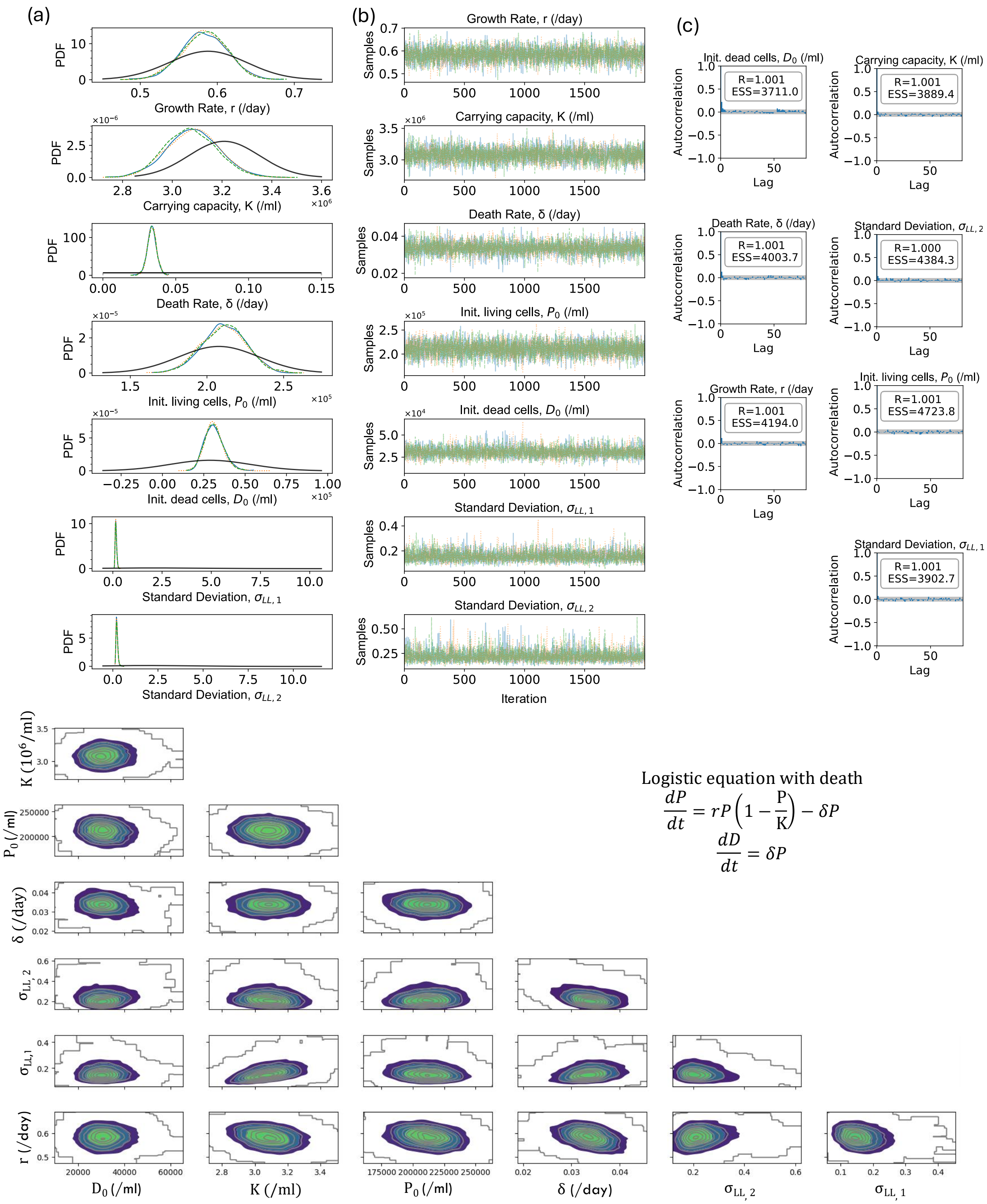

Figure S8: **Prior and Posterior statistics of fits with a logistic growth death model.** (a) Priors are shown in black lines, while the posterior kernel densities are plotted in colors for 3 different chains. (b) Trace plots for 3 independent chains for the sampled parameters. (c) Convergence statistics for all the chains combined. (d) Pairwise posterior plots for all variables. Growth rates and initial conditions exhibit strong negative posterior correlation, similar to Fig S3. Death rates are negatively correlated to carrying capacity of the algal system. Hyperparameters for this plot: Burn-in: 500 points for each chain, samples drawn: 2000 points for each chain, number of chains: 3, Thinning: None, Algorithm: NUTS.

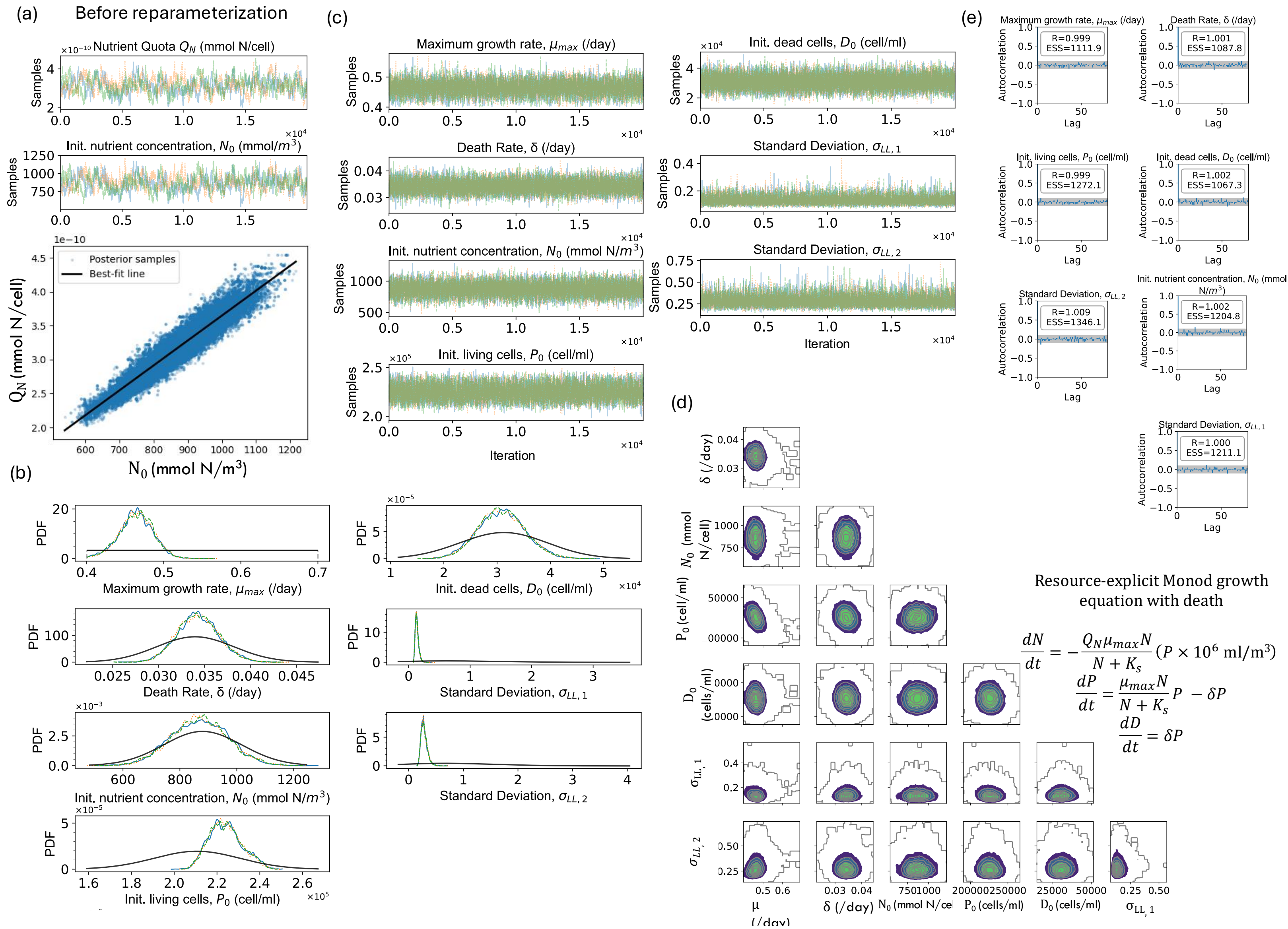

Figure S9: **Prior and Posterior statistics of fits with a Monod growth model.** (a) Before reparameterization, fitting both Q_N and initial nutrient concentration resulted in a positively correlated posterior where they are not identifiable together. We reparameterized Q_N in terms of N_0 in the model and fitted it to the data to get convergent chains. (b) Priors are shown in black lines, while the posterior kernel densities are plotted in colors for 3 different chains. (c) Trace plots for 3 independent chains for the sampled parameters. (d) Pairwise posterior plots for all variables. Growth rates and initial conditions exhibit strong negative posterior correlation, similar to FigS3. Death rates are negatively correlated with carrying capacity. (e) Convergence statistics for all the chains combined. Hyperparameters for this plot: Burn-in: 20000 points for each chain, samples drawn: 20000 points for each chain, number of chains: 3, Thinning: 50, Algorithm: Metropolis.

Case	Model	Prior and Likelihood ($\mathcal{L}$)	Hyperparameters
1.1	Exponential growth	$\mu \sim \mathcal{N}(0.5, 0.3)$ [2]<br>$P_0 \sim Log\mathcal{N}(\log(P_0^{obs}), 0.1)$<br>$\sigma_{LL} \sim Half\mathcal{N}(1)$<br>$\mathcal{L} \sim \mathcal{N}(\log(P^{model}) - \log(P^{data}), \sigma_{LL})$	burn-in per parameter per chain = 1000<br>samples per parameter per chain = 1000<br>chains = 4<br>target acceptance rate = 0.85<br>Sampler: NUTS
1.2	Exponential growth and death	$\mu \sim \mathcal{TN}(\mu = 0.5, \sigma = 0.3,$<br>upper bound $= 1$, lower bound $= 0.1)$ [2]<br>$\delta \sim \mathcal{TN}(\mu = 0.5, \sigma = 0.3,$<br>upper bound $= 1$, lower bound $= 0.1)$[20]<br>$P_0 \sim Log\mathcal{N}(\log(P_0^{obs}), 0.1)$<br>$\sigma_{LL} \sim Half\mathcal{N}(1)$<br>$\mathcal{L} \sim \mathcal{N}(\log(P^{model}) - \log(P^{data}), \sigma_{LL})$	burn-in per parameter per chain = 1000<br>samples per parameter per chain = 1000<br>chains = 4<br>target acceptance rate = 0.85<br>Sampler: NUTS
2.1	Logistic growth	$r \sim \mathcal{U}(0.5, 1)$[4]<br>$K \sim \mathcal{U}(10^6, 4 \times 10^7)$<br>$P_0' \sim \mathcal{N}(2.3 \times 10^5, 5 \times 10^4)$<br>$\sigma_{LL} \sim Half\mathcal{N}(3)$<br>$\mathcal{L} \sim \mathcal{N}(\log(P^{model}) - \log(P^{data}), \sigma_{LL})$	burn-in per parameter per chain = 1000<br>samples per parameter per chain = 2000<br>chains = 3<br>target acceptance rate = 0.95<br>Sampler: NUTS
2.2	Logistic growth with death	$r \sim \mathcal{N}(\mu_r^{Model2.1}, \sigma_r^{Model2.1})$<br>$K \sim \mathcal{N}(\mu_K^{Model2.1}, \sigma_K^{Model2.1})$<br>$\delta \sim \mathcal{U}(0, 0.15)$<br>$P_0 \sim \mathcal{N}(\log(T_0^{Model2.1} - D_0^{data}), \sigma_{T_0}^{Model2.1})$<br>$D_0 \sim Log\mathcal{N}(\log(D_0^{data}), \sigma_{T_0}^{Model2.1}))$<br>$\sigma_{LL,1} \sim Half\mathcal{N}(3)$<br>$\sigma_{LL,2} \sim Half\mathcal{N}(3)$<br>$\mathcal{L} \sim \mathcal{N}(\log(T^{model}) - \log(T^{data}), \sigma_{LL,1})$<br>$\times\mathcal{N}(\log(D^{model}) - \log(D^{data}), \sigma_{LL,2})$,<br>where $T = P + D$	burn-in per parameter per chain = 1000<br>samples per parameter per chain = 2000<br>chains = 3<br>target acceptance rate = 0.95<br>Sampler: NUTS
3	Resource explicit model<br>with Monod growth<br>(after reparameterization)	$\mu_{max} \sim \mathcal{U}(0.4, 0.7)$ [21]<br>$K_S \sim \mathcal{U}(0.05, 0.2)$[22]<br>$N_0 = \mathcal{N}(880, 100)$ [23]<br>Before reparameterization:<br>$Q_N \sim \mathcal{U}(2 \times 10^{-10}, 3 \times 10^{-9})$[24]<br>After reparameterization:<br>$Q_N = 3.67 \times 10^{-13} N_0 - 2.39 \times 10^{-12}$<br>$\delta \sim \mathcal{U}(0.01, 0.09)$<br>$P_0 \sim \mathcal{N}(\mu_{P_0}^{Model2.2}, \sigma_{P_0}^{Model2.2})$<br>$D_0 \sim \mathcal{N}(\mu_{D_0}^{Model2.2}, \sigma_{D_0}^{Model2.2})$<br>$\sigma_{LL,1} \sim Half\mathcal{N}(1)$<br>$\sigma_{LL,2} \sim Half\mathcal{N}(1)$<br>$\mathcal{L} \sim \mathcal{N}(\log(T^{model}) - \log(T^{data}), \sigma_{LL,1})$<br>$\times\mathcal{N}(\log(D^{model}) - \log(D^{data}), \sigma_{LL,2})$,<br>where $T = P + D$	burn-in per parameter per chain = 20000<br>samples per parameter per chain = 20000<br>chains = 3<br>Sampler: Metropolis Hastings

Table S1: **Priors and sampling hyperparameters for different growth models.** $\mathcal{U}$ and $\mathcal{N}$ denote Uniform distribution and Normal distribution respectively. For Uniform distribution the parameters are lower and upper bounds. For Normal distributions, the parameters are mean and standard deviations. For the Half-Normal distribution, the standard deviation of the underlying Normal distribution is noted. $\mathcal{N}(\mu, \sigma)$ refers to Normal distribution with μ and σ as the mean and standard deviation. $\mathcal{TN}$ refers to truncated Normal distribution where upper and lower bounds of the truncation are also specified. $\mathcal{U}(a, b)$ refers to a Uniform distribution bounded by a and b. $Half\mathcal{N}(1)$ refers to a half normal distribution with 1 as the scale parameter.

(a) Exponential growth models of *Phaeocystis globosa*

Model	Parameter	Description	Units	Mean ± SD
Exponential	μ	Growth rate	day^{-1}	0.281 ± 0.043
	N_0	Initial population	cells ml^{-1}	$(1.55 \pm 0.08) \times 10^6$
Exponential + death	μ	Growth rate	day^{-1}	0.655 ± 0.155
	δ	Death rate	day^{-1}	0.384 ± 0.154
	N_0	Initial population	cells ml^{-1}	$(1.56 \pm 0.09) \times 10^6$

(b) Logistic growth models of *Emiliania huxleyi*

Model	Parameter	Description	Units	Mean ± SD
Logistic	r	Growth rate	day^{-1}	0.588 ± 0.036
	K	Carrying capacity	cells ml^{-1}	$(3.21 \pm 0.10) \times 10^6$
	P_0'	Initial live population	cells ml^{-1}	$(2.36 \pm 0.18) \times 10^5$
Logistic + death	r	Growth rate	day^{-1}	0.583 ± 0.030
	δ	Death rate	day^{-1}	0.0336 ± 0.0032
	K	Carrying capacity	cells ml^{-1}	$(3.08 \pm 0.10) \times 10^6$
	P_0	Initial live population	cells ml^{-1}	$(2.11 \pm 0.14) \times 10^5$
	D_0	Initial dead population	cells ml^{-1}	$(3.11 \pm 0.58) \times 10^4$

(c) Monod growth with death model of *Emiliania huxleyi*

Parameter	Description	Units	Mean ± SD
K_s	Half-saturation constant	mmol N/m^3	0.34 ± 0.11
$\mu_{\max}$	Maximum growth rate	day^{-1}	0.464 ± 0.022
δ	Death rate	day^{-1}	0.033 ± 0.002
Q_N	Cellular nitrogen quota	mmol N/cell	$(3.16 \pm 0.37) \times 10^{-10}$
P_0	Initial live population	cells ml^{-1}	$(2.24 \pm 0.08) \times 10^5$
D_0	Initial dead population	cells ml^{-1}	$(3.08 \pm 0.44) \times 10^4$
N_0	Initial nutrient concentration	mmol N/m^3	870 ± 100

Table S2: Posterior mean ± standard deviation of model parameters inferred from Bayesian inference for different population dynamics models.

Supplementary Information References

[1] Ankit Rohatgi. *WebPlotDigitizer*. Version 5.2. Accessed on August 21, 2025. URL: https://automeris.io.

[2] A-C Baudoux and Corina PD Brussaard. "Characterization of different viruses infecting the marine harmful algal bloom species *Phaeocystis globosa*". In: *Virology* 341.1 (2005), pp. 80–90.

[3] T Castberg et al. "Microbial population dynamics and diversity during a bloom of the marine coccolithophorid *Emiliania huxleyi* (Haptophyta)". In: *Marine Ecology Progress Series* 221 (2001), pp. 39–46.

[4] Noa Barak-Gavish et al. "Bacterial virulence against an oceanic bloom-forming phytoplankter is mediated by algal DMSP". In: *Science advances* 4.10 (2018), eaau5716.

[5] Stan Development Team. *Stan Modeling Language Users Guide and Reference Manual*. 2023. URL: https://mc-stan.org.

[6] Tor Erlend Fjelde et al. "Turing.jl: a general-purpose probabilistic programming language". In: *ACM Transactions on Probabilistic Machine Learning* (Feb. 2025). ISSN: 2836-8924.

[7] Travis E Gibson et al. "Learning ecosystem-scale dynamics from microbiome data with MDSINE2". In: *Nature Microbiology* (2025), pp. 1–15.

[8] Oriol Abril-Pla et al. "PyMC: a modern, and comprehensive probabilistic programming framework in Python". In: *PeerJ Computer Science* 9 (2023), e1516.

[9] Hong Ge, Kai Xu, and Zoubin Ghahramani. "Turing: a language for flexible probabilistic inference". In: *International conference on artificial intelligence and statistics*. PMLR. 2018, pp. 1682–1690.

[10] Greg Brunkhorst. "ODE Lotka-Volterra With Bayesian Inference in Multiple Ways. In: PyMC Examples". In: *PyMC examples*. Ed. by PyMC Team. DOI: 10.5281/zenodo.5654871.

[11] Turing.jl Developers. *Bayesian Differential Equations Tutorial*. https://turinglang.org/docs/tutorials/bayesian-differential-equations/. Accessed: 2026-02-19. 2025.

[12] Lauren C. Ponisio et al. "One size does not fit all: Customizing MCMC methods for hierarchical models using NIMBLE". In: *Ecology and Evolution* 10.5 (Feb. 2020), pp. 2385–2416. ISSN: 2045-7758.

[13] Nicolaas Godfried Van Kampen. *Stochastic processes in physics and chemistry*. Vol. 1. Elsevier, 1992.

[14] Raunak Dey et al. "Emergent higher-order interactions enable coexistence in phage-bacteria community dynamics". In: *bioRxiv* (May 2025).

[15] Stephen McCoy et al. "Quantitative Assessment of Biological Dynamics with Aggregate Data: S. McCoy et al." In: *Bulletin of Mathematical Biology* 87.11 (2025), p. 164.

[16] Stephen J. Beckett et al. "Disentangling top-down drivers of mortality underlying diel population dynamics of *Prochlorococcus* in the North Pacific Subtropical Gyre". In: *Nature Communications* 15.1 (Mar. 2024). ISSN: 2041-1723.

[17] David Demory et al. "Linking Light-Dependent Life History Traits with Population Dynamics for *Prochlorococcus* and Cyanophage". In: *mSystems* 5.2 (Apr. 2020). Ed. by Steven J. Hallam. ISSN: 2379-5077.

[18] Matthew D Hoffman, Andrew Gelman, et al. "The No-U-Turn sampler: adaptively setting path lengths in Hamiltonian Monte Carlo." In: *J. Mach. Learn. Res.* 15.1 (2014), pp. 1593–1623.

[19] Radford M Neal et al. "MCMC using Hamiltonian dynamics". In: *Handbook of markov chain monte carlo* 2.11 (2011), p. 2.

[20] L Peperzak et al. "Growth and mortality of flagellates and non-flagellate cells of *Phaeocystis globosa* (Prymnesiophyceae)". In: *Journal of Plankton Research* 22.1 (2000), pp. 107–120.

[21] Rosie M Sheward et al. "Elemental stoichiometry of the key calcifying marine phytoplankton *Emiliania huxleyi* under ocean climate change: A meta-analysis". In: *Global Change Biology* 29.15 (2023), pp. 4259–4278.

[22] Richard W Eppley, Jane N Rogers, and James J McCarthy. "Half-saturation constants for uptake of nitrate and ammonium by marine phytoplankton 1". In: *Limnology and oceanography* 14.6 (1969), pp. 912–920.

[23] Robert RL Guillard. "Culture of phytoplankton for feeding marine invertebrates". In: *Culture of marine invertebrate animals: proceedings—1st conference on culture of marine invertebrate animals greenport*. Springer. 1975, pp. 29–60.

[24] Mónica Rouco et al. "The effect of nitrate and phosphate availability on *Emiliania huxleyi* (NZEH) physiology under different CO_2 scenarios". In: *Frontiers in microbiology* 4 (2013), p. 155.
